# PADL: A Modeling and Deployment Language for Advanced Analytical Services †

**DOI:** 10.3390/s20236712

**Published:** 2020-11-24

**Authors:** Josu Díaz-de-Arcaya, Raúl Miñón, Ana I. Torre-Bastida, Javier Del Ser, Aitor Almeida

**Affiliations:** 1 TECNALIA, Basque Research & Technology Alliance (BRTA), 48160 Derio, Spain; raul.minon@tecnalia.com (R.M.); isabel.torre@tecnalia.com (A.I.T.-B.); javier.delser@tecnalia.com (J.D.S.); 2 Department of Communications Engineering, Faculty of Engineering, University of the Basque Country (UPV/EHU), 48013 Bilbao, Spain; 3 DeustoTech, University of Deusto, Avenida de las Universidades 24, 48007 Bilbao, Spain; aitor.almeida@deusto.es

**Keywords:** edge computing, analytical pipelines, machine learning life cycle, artificial intelligence description language

## Abstract

In the smart city context, Big Data analytics plays an important role in processing the data collected through IoT devices. The analysis of the information gathered by sensors favors the generation of specific services and systems that not only improve the quality of life of the citizens, but also optimize the city resources. However, the difficulties of implementing this entire process in real scenarios are manifold, including the huge amount and heterogeneity of the devices, their geographical distribution, and the complexity of the necessary IT infrastructures. For this reason, the main contribution of this paper is the PADL description language, which has been specifically tailored to assist in the definition and operationalization phases of the machine learning life cycle. It provides annotations that serve as an abstraction layer from the underlying infrastructure and technologies, hence facilitating the work of data scientists and engineers. Due to its proficiency in the operationalization of distributed pipelines over edge, fog, and cloud layers, it is particularly useful in the complex and heterogeneous environments of smart cities. For this purpose, PADL contains functionalities for the specification of monitoring, notifications, and actuation capabilities. In addition, we provide tools that facilitate its adoption in production environments. Finally, we showcase the usefulness of the language by showing the definition of PADL-compliant analytical pipelines over two uses cases in a smart city context (flood control and waste management), demonstrating that its adoption is simple and beneficial for the definition of information and process flows in such environments.

## 1. Introduction

In recent years, the concept of smart cities has emerged in response to the challenges posed by the continuous development of urban infrastructures and the increase in population density. The main objective of this paradigm is to enhance the management of the city by providing smarter, safer, and more sustainable ecosystems [1]. In order to properly address these challenges, specific services and systems have to be developed and provided to citizens. This leads to an improvement of their quality of life; on the other hand, more complex ICT infrastructures become necessary.

In this context, Artificial Intelligence (AI) is a key enabling technology, since it provides the necessary foundations for the intelligence and resilience of future cities. Furthermore, ICT tools can deal with the diverse application domains that exist in a city. For instance, Lim et al. [2] conducted an intensive study of the related literature to identify twelve distinct domain categories: “smart device”, “smart environment”, “smart home”, “smart energy”, “smart building”, “smart transportation”, “smart logistics”, “smart farming”, “smart security”, “smart health”, “smart hospitality”, and “smart education”. These categories share the objective of building an integrated and advanced intelligent information ecosystem to enable a framework that will boost the socioeconomic growth of the city. However, extracting valuable information from the collected data produced requires effective techniques, tools, and software technologies to collect, store, analyze, and visualize large amounts of data from the city environment, its organizations, departments, agencies, and citizens.

Some authors [3] have stated that the ideal model of a smart city is mainly based on the following pillars, in which AI plays a key role:

1. Smart sensors enable the collection of the necessary data and transform the city into a smart city. They maintain the city connected and the stakeholders informed, and without them, the rest of subsystems would not be able to function correctly.
2. Smart citizens are undoubtedly the fundamental part of a smart city since their active involvement makes it possible to perform these initiatives.
3. Smart services enable citizens and administrative entities to actively participate in the control of the city. They are based on information technologies that help to control the different subsystems that comprise the smart city.

In isolation, these statements present challenges that have to be addressed. Nevertheless, the bottom line is offering a unified solution to enable a coordinated and effective flow of information (both from sensors and citizens) and the execution of processes among the potential advanced services offered by the city. Next, the different pillars and the problems detected by each one are introduced in detail.

The growing adoption of sensors being deployed in cities, smart sensors, has been significantly favored by certain paradigms such as the Internet of Things (IoT). This paradigm expands the definition of smart cities, which become more connected. However, this innovation will largely depend on the capabilities of the underlying technologies to leverage the information and resources supplied by the vast volume of emerging devices. Therefore, the key to achieve intelligent cities depends on the adequate analysis of the connected data sources. In addition, IoT devices offer the possibility of lowering the computations down to the edge layer, that is to say, closer to where data are generated and collected. This can be particularly attractive in contexts where the latency caused by the interactions among the elements of the IoT environment and the cloud is too high [4] or crucial. These two technological advances, a greater distribution of knowledge and its computation and the appearance of more advanced sensors capable of executing certain computations reducing the latency, must be included in the services proposed by cities. This is because in spite of presenting a great technological complexity, they can significantly improve the final experience of citizens.

Currently, knowledge, computing, and storage infrastructures tend to be distributed. This implies that systems, architectures, algorithms, and techniques designed for cities have to face additional requirements when being implemented (e.g., scalability, partitioning, and distribution).

The smart city concept lies at the intersection of city administration, citizen value creation, local business, ICT development and application, urban Big Data, economics, and sociology, citizen engagement (smart citizens) being a key element of most definitions of smart cities. Additionally, Information and Communication Technologies (ICTs) play a crucial role as the drivers and enablers of citizen participation, being the necessary technologies for processing the information with AI and providing in this way a better service to the relevant actors. In this context, it is essential that new services have functionalities like self-monitoring in order to guarantee their stability and correct maintenance. On the other hand, users need to interact appropriately with the smart city ecosystem. Therefore, it is necessary to provide services with the possibility of sending notifications, alerts, and mechanisms for actuating on the devices.

In recent times, the continuous advances in AI have revolutionized intelligent solutions in a plethora of domains. Specifically in the smart city domain, it becomes clear that AI must be the cornerstone to generate new valuable services, the formerly called smart services. The main areas of improvement in which AI affects cities are efficient resource management [5] and the improvement of the citizens’ quality of life [6]. Regarding the data being generated by smart cities, two main considerations must be taken into account: its massive volume, due to the growing generation of data by citizens and devices, and its sparseness, due to the ubiquitous deployment of various types of sensors. These characteristics require a new computing paradigm to offer location-aware and latency-sensitive monitoring and intelligent control [7].

As a result of this new architectural landscape, new AI approaches have arisen in recent times, with a manifold of implications in terms of operationalization: (i) federated learning is a distributed machine learning paradigm that enables privacy-aware learning over distributed data by using edge computing and (ii) stream learning techniques on the edge, in which nearly real-time learning algorithms can be deployed closer to IoT devices for lower latencies, resiliency, efficient use of bandwidth, and compliance. This work indeed addresses this particular strand of challenges, which is collectively referred to as “operationalization” since the proposed language does not only focus on the definition, but also includes functionalities for the deployment and monitoring of analytical services.

However, the scientific community working in the fields of AI and Data Science (DS) has mainly been focused on the development of new algorithms and learning techniques, in many cases under laboratory or experimental setups. Consequently, transferring these prototypes and tests to real production environments, as is very often demanded by smart city stakeholders, has proven to be difficult to achieve with those approaches. This article stresses this vision, namely the way in which smart data-based services are provisioned in real infrastructures.

In this work, a proposal to tackle this is provided from the ICT perspective: A description language that eases the definition and operationalization of these data flows and the associated processes. By virtue of this language, the difficulties emerging when working with complex disciplines such as Big Data, AI, or IoT, alongside those explained above: (i) infrastructure distribution, (ii) AI operationalization, and (iii) the large number and ubiquity of sensors, can be minimized. Summarizing, this manuscript revolves around the different needs presented by the services and use cases that fall within the smart city domain, such as: (a) the definition of complex and composite analytical services through different phases in which various actors and data sources can be involved; (b) the deployment of these services in the form of analytical pipelines on very diverse ICT infrastructures (i.e., edge, fog, and cloud); and (c) the provision of heterogeneous actuation mechanisms, using the devices and sensors (actors), in the form of alerts or actuators. As a consequence, the main contribution of this article is a domain specific language coined as the Analytical Pipeline Definition and Deployment Language (PADL), which is devoted to supporting the operationalization of analytical pipelines in heterogeneous infrastructures by enabling the definition and provision of complex analytical services in the domain of smart cities. In addition, data scientists do not need to fathom the infrastructure and underlying technologies in detail; instead, they can use annotations so the models are operationalized appropriately, in a reproducible manner. Finally, certain tools for the smooth deployment of analytical pipelines in production environments are presented.

The proposed language is validated using two use cases in the domains of flood control and waste management. In both cases, we exemplify how to use PADL to define the pipeline and the technical complexities in detail, as well as the benefits it brings to the city ecosystem (ICT infrastructure and actors). To the best of our knowledge, there are no languages or tools similar to the one proposed in this article. Therefore, instead of comparing its efficiency against other works, the demonstrations focus on highlighting its usefulness.

The rest of the paper is organized as follows. Section 2 presents related work in the scope of provisioning analytic services in smart cities and technologies related to the deployment in the production of analytical processes. Section 3 describes the main features of the proposed language PADL. Section 4 elaborates on the technical details of the language implementation, along with a set of auxiliary tools that assist in its use. Section 5 presents the two use cases, which serve as the validation of the practical utility of the PADL language. Finally, conclusions and future work are presented in Section 6. A preliminary study, which did not include the implementation and tools provided in this paper and was not specifically focused on the smart city domain, was presented as a conference paper [8].

## 2. Deploying Data Analytics in Smart Cities

Different software solutions have been proposed in the literature to provide smarter, safer, and more sustainable cities through new paradigms like Big Data, AI, or IoT. The main objective of these solutions is to generate innovative services to citizens leveraging AI techniques. This section is divided into two parts: the first introduces different research projects and initiatives that deal with advanced analytical services that can be integrated into the city ecosystem; the second part explains the technological background necessary to understand the difficulties of deploying these solutions and services in production environments.

### 2.1. Analytic Services for Smart Cities

There has been an enormous increase in the data generated in urban ecosystems. These data can be useful for providing high-value services in different domains by using analytical techniques. Arasteh et al. [9] provided a review of the concept of smart cities, its motivations, and applications. Additionally, they examined IoT technologies for smart cities and their main components and features. Sanchez et al. [10] performed a study on the future technological challenges of smart cities, where they pointed out the complexity of the analytical process flows needed in this context. For instance, in the smart city domain, the work in [11] proposed a Big Data-driven analysis and a cloud-based analytic service that utilizes urban environment indicators such as quality of life. Hossain et al. in [12] analyzed GIS data, historical data, and other parameters such as building slope, flow accumulation, land use, soil types, and distance from the river in order to explore individual residential and business buildings for flooding risk in Birmingham (U.K.). Mazhar et al. [13] proposed a system with various types of sensors ranging from smart homes, vehicular networking, and weather, water, and smart parking sensors, surveillance objects and a four-tier architecture devoted to manage IoT sources, communications, and data, using Big Data technologies for processing and serving data. This way, they analyzed both in batch and real-time smart homes, smart parking, weather, pollution, and vehicle datasets aiming at optimizing the urban planning and enhancing the future city development. These types of approaches could be potentially simplified by using the PADL syntax, since it provides an abstraction layer over the underlying infrastructure deployment and eases the analytical pipeline conceptualization.

Citizens are one of the pillars of smart cities, and the article presented by Aguilera et al. [14] focused on how to accelerate the generation of citizen-centric apps that exploit urban data in different domains. Furthermore, there are very interesting studies that try to take advantage of citizen potential (data and participation) through applications, based on IoT or AI, and by means of initiatives like citizen science. The study proposed by Lopez-Novoa et al. [15] is a clear example, explaining how to use an IoT communications platform to promote a citizen science project, where individuals collect, categorize, and sometimes analyze scientific data. Furthermore, there are studies like [16] that acknowledged the huge amount of information citizens are exposed to and proposed a cognitive-driven, personalized information system to minimize potential cognitive overload issues.

In addition to the previous works that offered overhauls on the benefits of analytical data in the context of smart cities, there are currently many works focused on solving very specific problems or use cases in the smart city domain, like that presented by Cerchecci et al. [17] for optimizing waste collection or that presented by Obinikpo et al. [18] for smarter health care in smart cities. Additional literature in the field of smart city analytics was explored by Wang et al. in [19], where a survey of deep learning techniques in smart cities was offered. Concretely, it examined algorithms applied to video analytics of smart cities in terms of different research categories: object detection, object tracking, face recognition, image classification, and scene labeling. Moreover, Saggi et al. in [20] deeply explored the concept of Big Data analytics, and a methodical analysis for the usage of Big Data analytics in various domains including smart cities was presented. Lavalle et al. in [21] proposed a methodology based on visualization techniques, which assist users, the goal being to improve the evidence-gathering process, in this way contributing to the optimization of the city resources.

Urban mobility is yet another field of study in smart cities. Vergis et al. in [22] proposed a low cost traffic monitoring system using IoT devices and fog computing and provided a system capable of estimating traffic flow, which can be exploited by the authorities. Tekouabou et al. [23] proposed a system that integrates IoT and set-based regression models to predict the availability of free places for parking, hence reducing urban congestion. Hîrţan et al. in [24] presented a reputation system with the purpose of providing users with an optimal travel route, focusing at the same time on privacy and confidentiality.

However, all these works focus on building dedicated systems over a specific infrastructure. The approach presented in this article is positioned at a higher level of abstraction from the ICT perspective. The main idea is to provide an analytical service definition and deployment language in the domain of smart cities. This way, these definitions can be integrated with the existing city ecosystem/model and ICT infrastructure. Additionally, each new advanced analytical service does not have to be conceptualized as a particular solution or independent system, but be defined appropriately according to the proposed language aiming at being deployed in the existing infrastructure.

### 2.2. Technological Background

There are two main approaches in the context of the deployment of analytical pipelines in production environments:

- Deployment frameworks: In this field, there are novel works such as MLflow [25], which provides tools for packaging and deploying analytical pipelines in different environments. ML.NET [26] is another open-source framework proposed by Microsoft to deploy machine learning pipelines. For typical operational workflow operationalization, on the other hand, Verma et al. [27] proposed a cluster manager to execute hundreds of workloads across thousands of machines. This project was the cornerstone for Kubernetes [28]. Another effort towards the orchestration of workloads in distributed environments is Docker Swarm [29], which offers less features than Kubernetes, but at a lower technological footprint. However, both Kubernetes and Docker Swarm are general-purpose deployment frameworks and do not focus specifically on ML or AI.
- Description languages: In the machine learning domain, the Predictive Model Markup Language (PMML) [30] was one of the first solutions that tackled the problems associated with the operationalization of AI and ML models. Subsequently, Portable Format for Analytics (PFA) [31] was conceptualized claiming that, in contrast to PMML, it is an extensible language for the definition of pre-processing and post-processing code, provides better features to create analytical workflows, integrates easily with distributed and event-based data processing platforms, and is safer to use within IT operational environments.

PADL is conceived of to coexist at the intersection of these two approaches, with a twofold goal: first, to be specific to the analytical scope and to understand its nuances; and secondly, to be sufficiently expressive, providing a means for the deployment of these pipelines in heterogeneous environments, technologies, and infrastructures. Table 1 offers a comparison of the aforementioned technologies in conjunction with some trends found in academia, and all of them are evaluated against the criteria explained below:

1. Deployment awareness, allowing for the definition of the restrictions an analytical pipeline needs to adhere to when deployed in production.
2. Domain orientation, providing annotations specific for the analytical domain.
3. Interacts with technologies already existing in the infrastructure (i.e., Apache Spark [32], Apache Flink [33]).
4. Permits the definition of the entire pipeline alongside the annotations in a single text file, so it can be versioned and integrated into continuous integration and delivery workflows.
5. Has a low technological footprint, facilitating the use of existing infrastructures with heterogeneous devices.
6. Enables the deployment of analytical pipelines in different layers of the architecture (i.e., edge, fog, cloud).

Both PMML and PFA excel at the formalization of analytical pipelines, but fail in terms of integration with existing technologies. Additionally, they do not contemplate the deployment over different computation layers, nor do they consider distributed processing. On the other hand, Kubernetes and Docker Swarm are proficient at deploying ecosystems at multiple nodes, but are general-purpose tools; hence, they lack the ability to understand analytical pipelines towards distributing them properly. Finally, MLflow and ML.NET shine in training and packaging analytical pipelines, but only cover the deployment to some extent, without being able to fully utilize the existing technologies, nor being aware of the various computational layers (e.g., edge, fog, cloud). Among the most recent works, we do not find similar approaches, and we can only review the Stratum and DEEP-Hybrid-DataCloud frameworks. Stratum [34] focuses on analytics, but it is not text based nor does it focus on multilayered deployments. The DEEP-Hybrid-DataCloud [35] framework is very similar to Stratum, and it lacks the awareness for being deployed in multiple processing layers. Summarizing, currently, there is no tool that can efficiently compete in all the different aspects of the operationalization of data-based pipelines. Those of the analytical domain do not easily adopt the benefits of distributed environments. Conversely, deployment tools lack the capacity of understanding analytical pipelines and cannot parallelize them effectively across edge and fog environments.

## 3. PADL Specification

Our proposed PADL domain specific language enables the definition of analytical pipelines and provides a specific syntax for enriching it with functional and non-functional requirements. Among other features, PADL: (i) abstracts the user from the underlying infrastructure and technologies; (ii) enables the deployment of the analytical pipeline in various computational layers (edge, fog, cloud); (iii) promotes the operationalization of these pipelines over continuous integration and deployment environments; (iv) provides a means for specifying performance, security, and monitoring capabilities alongside its definition; and (v) simplifies the development process, since data scientists can test the models in different infrastructures before deploying them in production. PADL is a fresh twist on the everything-as-code [36] trend and can be written/edited with a simple text editor in YAML [37] format, which makes it readable by humans and machines. Documents written in PADL should be validated against a schema that can be found in the official PADL repository [38].

### 3.1. Ecosystem

An architecture that reconciles various computing layers within a Big Data paradigm is proposed by Díaz-de-Arcaya et al. in [39]. This architecture expands across the edge, fog, and cloud computing layers and is oriented toward the deployment of analytical pipelines in heterogeneous infrastructures. Among the assorted components that integrate this architecture, the focus of this research work is to provide a basic building block for the implementation of the Analytic Orchestrator and Life Cycle Management module, a domain specific languagecalled PADL. This component is able to understand a PADL document and deploy the defined analytical pipeline in the infrastructure. PADL, on the other hand, serves as the definition for the analytical pipeline and offers annotations to abstract the user from the shortcomings of production deployments (e.g., infrastructure details, network constraints, technologies).

**Figure 1:**
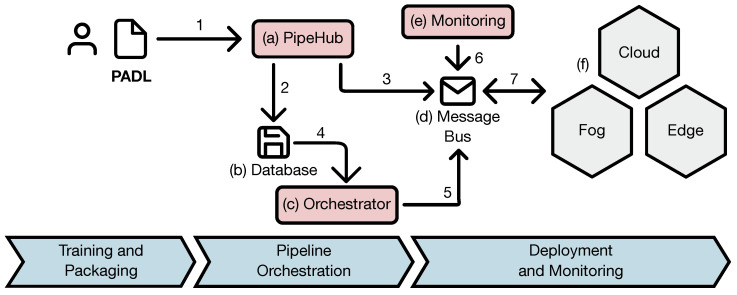
Reference PADL architecture.

The PADL deployment flow is schematically depicted in Figure 1. It comprises three stages: (i) training and packaging, (ii) pipeline orchestration, and iii) deployment and monitoring. Initially, the data scientist builds his/her analytical pipeline using his/her preferred library and packages the different models either using a definition language (e.g., PFA [31], PMML [30]) or a machine learning specific packaging system (e.g., MLflow Projects (https://mlflow.org/docs/latest/projects.html)). Then, he/she creates a PADL document with the definition of the analytical pipeline and enriches it with deployment specific annotations (i.e., how the pipeline should be deployed in the production environment) and machine learning related annotations (i.e., which action should be taken if the performance of a model drops below a certain threshold). The data scientist sends (1) the model over to the architecture, where the (a) PipeHub module persists (2) both the trained models and the PADL document in a (b) database. In addition to this, it sends (3) a message to the (c) Orchestrator through the (d) Message Bus, which is the module in charge of the communications across the whole architecture, and with the infrastructure (7). Then, the Orchestrator retrieves (4) a document from the database with information related to the infrastructure in which the pipeline will be deployed. At this point, the Orchestrator has both the PADL and the infrastructure documents and is capable of making a decision on where each stage of the pipeline will be deployed in the production environment. This information will be communicated (5) to the infrastructure through the (d) Message Bus. Finally, the (e) Monitoring module ensures (6) that the pipeline is deployed and operates according to the annotations. Should an anomaly occur, a notification is sent to the stakeholders through a predefined channel.

The devices that conform the infrastructure use an agent for communicating with the architecture. This agent publishes the capacities of the node, so that a map of the whole infrastructure can be built. The communication between the Orchestrator and the infrastructure is accomplished through a publish-subscribe protocol (e.g., MQTT, Kafka, CoAP [40]). This technology has been chosen because, especially in the lower layers (i.e., edge), a single model may be deployed in thousands of devices; relying on having to send the same message across the network to that many devices would not be feasible. On the contrary, a publish-subscribe protocol would only require the message to be published in a single topic to be spread across the network.

In a smart city context, the PADL language can offer additional value since analytical pipelines can rapidly be defined and deployed without having to modify the existing infrastructure. This benefit enables modern cities to easily provide new services to citizens and to be able to adapt to new contexts in an agile way. In addition, it is worth mentioning that due to the monitorization, notification, and actuation capabilities supported by PADL, as soon as a specific event in the city is identified, a notification mechanism can be triggered and inform the relevant stakeholders. In addition, specific actuators can be managed by PADL in order to address the problem.

### 3.2. Language Details and Application Preconditions

The Analytical Pipeline Definition and Deployment Language (PADL) is a domain specific language for the description of analytical pipelines, including a definition level intended for their operationalization that covers the deployment characteristics necessary to implement them on previously defined and available infrastructures. A data analytic pipeline is comprised of different data analysis phases, materialized in a compound of command-line tools and custom scripts that implement each of these analytic processes. For instance, an analytical pipeline could be used to detect traffic jams and react accordingly through suitable mobility plans. In this context, PADL would act as the framework for defining all the necessary analytical stages, from the processing of vehicle GPS signals, to decision-making in the form of alternative routing to be used by citizens. Furthermore, it allows the automation of all the processes involved in existing heterogeneous IT infrastructures.

**Figure 2:**
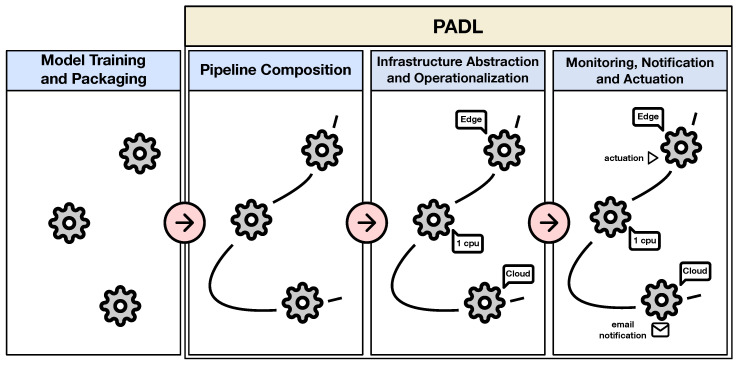
Definition and operation of analytical pipelines through the functionalities provided by PADL.

PADL is a description language that assists in the deployment of analytical pipelines considering very heterogeneous production environments. It provides a means for the definition of domain agnostic analytical pipelines, as well as for the specification of the automation and operationalization criteria of the implementation processes. Figure 2 showcases the definition process of analytical pipelines via PADL and how this language enables the enrichment of the analytical models and data processing stages, by utilizing the available features for the operationalization. At first, the data scientist trains his/her models and packages them in a certain format. Afterwards, PADL queues promote the definition of a flow with these models. Then, models can be be deployed in different layers (e.g., edge, cloud) and be constrained to execute under different infrastructure conditions (e.g., two CPU cores, 8GB RAM), all this with no knowledge of the infrastructure by the data scientist. Finally, watches enable the definition of actuators and notifications depending on the desired monitoring conditions.

PADL is intended to be a simple, normalized, and interoperable language; therefore, it has been built as a subset of the YAML serialization language, with a relatively simple syntax and designed to be comprehensible. The pipeline stages and their characteristics can be adequately represented as combinations of lists, mappings, and scalar data (simple values). This way, any library or analytical platform willing to utilize PADL will need to import and export its definitions in this simple format.

**Figure 3:**
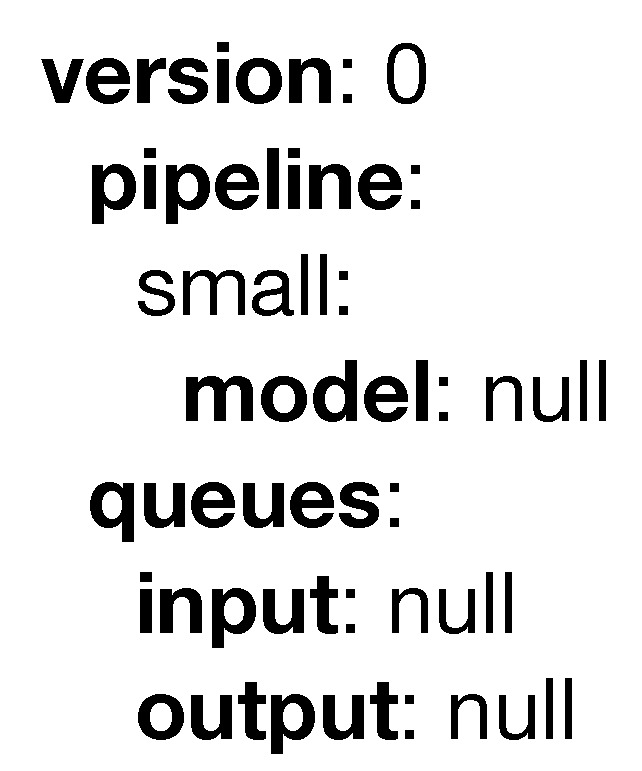
Minimum example of a PADL document.

The simplest example of PADL is that composed of an empty pipeline, as can be seen in Figure 3.

In Section 3.3, an extended syntax explanation is provided, in which each of the elements that comprises a pipeline is explained in further detail.

Finally, it is important to establish the application conditions to be met and the main objectives to be achieved through the use of PADL. The application conditions are only two: (i) the models of each stage of the analytical pipeline must have been previously selected, trained, and packaged in order to be specified in PADL; (ii) the provisioning and configuration of the infrastructure are not the responsibility of PADL; it will only be in charge of defining the restrictions and characteristics that should be met for the operationalization to be successful. The main PADL objectives orbit around the following points:

1. It should aspire to become a standard for the definition of analytical process flows.
2. It should help data analysts and domain experts overcome the technological barriers that prevent greater success in the operationalization of analytical processes.
3. It must be expressive enough to allow the definition of analytical pipelines as a chain of processing stages, in which each stage represents a model and its characteristics.
4. It must be able to be easily integrated into different analytical platforms and systems responsible for the operation of large analytical workloads.

### 3.3. Language Syntax

**Figure 4:**
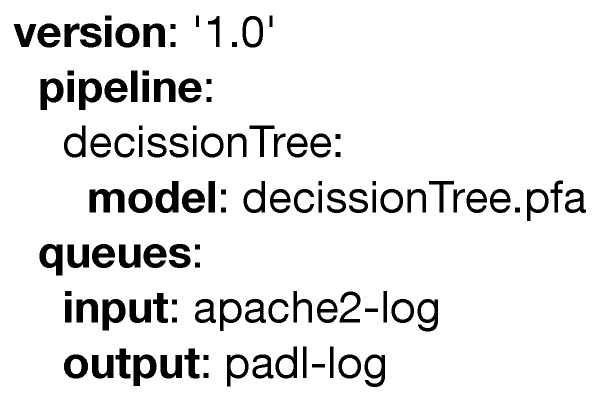
Simple example of a PADL document.

In this subsection, a simple PADL document is used to introduce the language. Then, the complete specification is discussed in further detail. The PADL document portrayed in Figure 4 specifies an analytical pipeline composed of a single model that has been packaged using the Portable Format for Analytics language.

The pipeline in Figure 4 will deploy the model *decissionTree.pfa* into the infrastructure. As there are no other attributes defined for this particular pipeline, we will assume the existing elements already fulfill the requirements for its execution. This model will monitor a text file, in this case the logs generated by an existing web server, and will populate another text file with the results.

**Figure 5:**
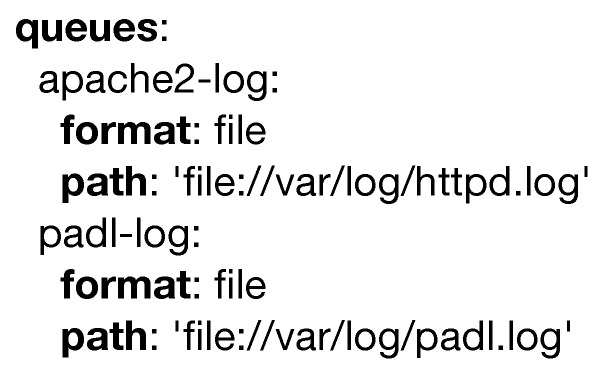
Example of the queues keyword.

Even though this is a very simple example, it introduces the use of queues as it can be seen in Figure 5. The purpose of this code structure is to specify the inputs and outputs for the different models. For the sake of simplicity, reading and writing to a text file is herein considered, yet a myriad of other different possibilities exist.

**Figure 6:**
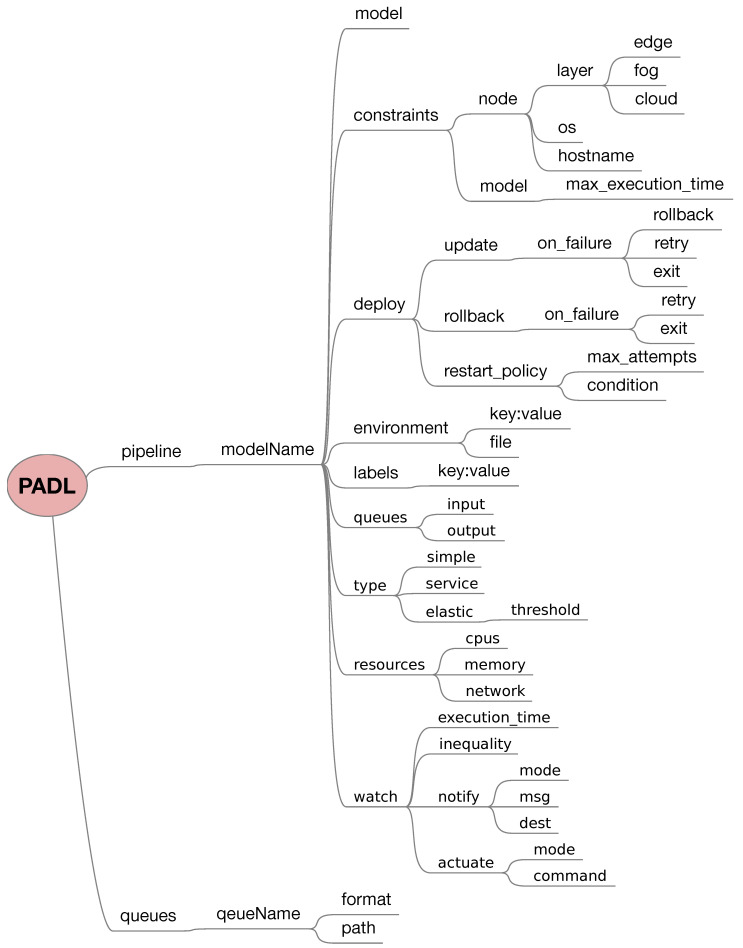
Tree diagram showing the PADL schema.

The complete PADL schema can be observed in Figure 6, in which pipeline and queue structures were discussed previously. These are the most important entities within a PADL document, which should contain both. The former defines which data transformation processes or models need to be run as part of the pipeline, whereas the latter defines how these models communicate with their environment and among themselves.

**Figure 7:**
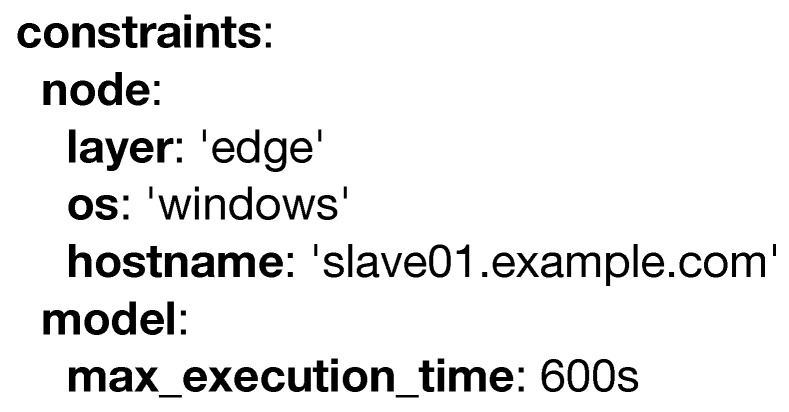
Example of the constraints keyword.

Another important entity is constraints which can be seen in Figure 7. This structure may have two children: (i) the **node** attribute specifies variables such as the operating system, the hostname, or the layer in which the model must run (e.g., this model must only be run in the edge layer); and (ii) the **model** itself, which defines constraints specific to the machine learning domain, such as **max_execution_time**, which defines the maximum time granted for a model to produce its output (e.g., a predicted value of the target variable in predictive modeling), beyond which it will halt its execution.

**Figure 8:**
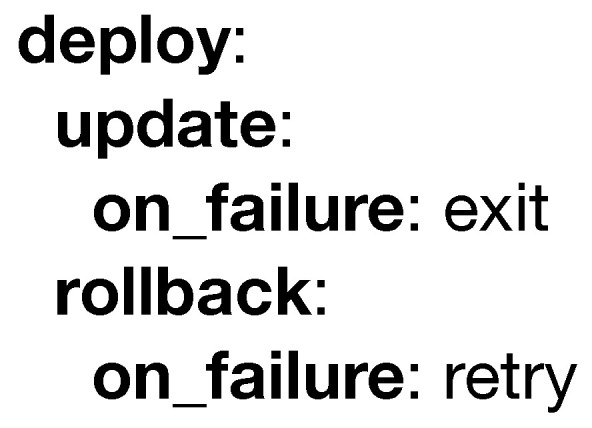
Example of the deploy keyword.

In Figure 8 the **deploy** structure determines the behavior during an update (i.e., in case the efficiency of the deployed model has decreased and needs to be redeployed) and during a rollback (i.e., the previous deployment is not performing as expected, so that a previous version will be deployed). Both cases support the **on_failure** attribute. In the case of a rollback failure, the counteracting behavior can be to declare the model as deprecated and stop trying to redeploy it, or to keep trying until it succeeds. However, an update supports both **retry** and **exit** attributes in the case of failure, but it also supports the **rollback** of the model to the previous version.

Another children of the model structure is the **environment** attribute. This differs from the previous ones in which is not constrained to a predefined set of attributes, but it can hold any key-value pair. It is commonly used for specifying variables the model will have to use in order to meet its purpose (e.g., specifying the full path to the version of Java the model needs). In addition to this, instead of having to write all the variables one at a time, it accepts a file path, so all variables can be added to that file and shared across the whole pipeline.

**Figure 9:**
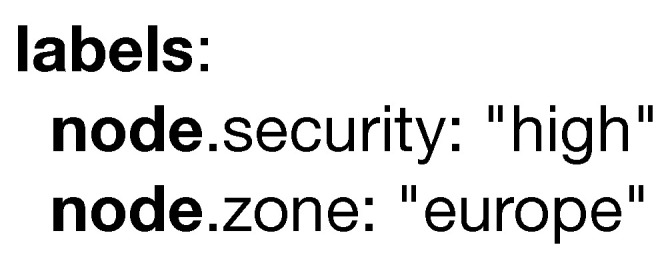
Example of the labels keyword.

**Labels** is another keyword that behaves similarly to the **environment** keyword, in which it can accept any amount of non-predefined key-value pairs. A model annotated with a label will match the devices in the infrastructure annotated with the same label. This is useful to deploy a model in a geographical location (e.g., legislation differs from European to American servers) or to deploy a model in a device subject to high security policies. An example of it can be seen in Figure 9.

**Figure 10:**
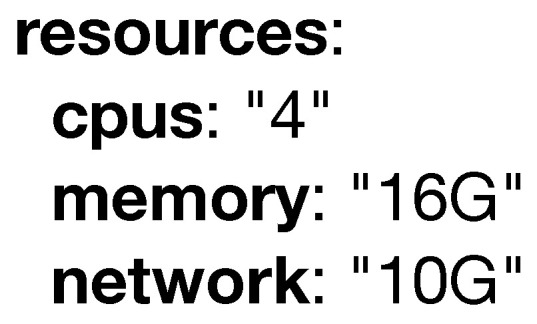
Example of the resources keyword.

The **resources** attribute, which can be seen in Figure 10 is used to specify the system requirements (e.g., CPU, memory) reserved by a certain model to fulfill its task. The *Orchestrator* depicted in Figure 1 can use this information to guarantee no device is planned over its resource limits.

**Figure 11:**
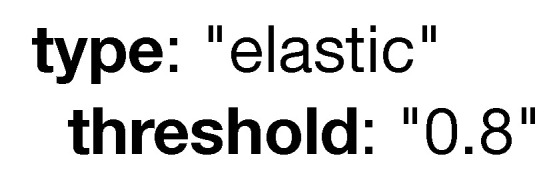
Example of the type keyword.

The **type** attribute, which is portrayed in Figure 11, defines how a model is deployed in production. Marking a model as simple has no impact, and is deployed once in the infrastructure. Type service, on the other hand, means a copy of the model will be deployed on all the devices that fulfill the specified criteria. Lastly, the elastic type represents a model that will scale up or down based on system load (i.e., if the model is using more than 80% of the system resources, another instance of the pipeline is created and deployed on another device that fully complies with the specified constraints).

**Figure 12:**
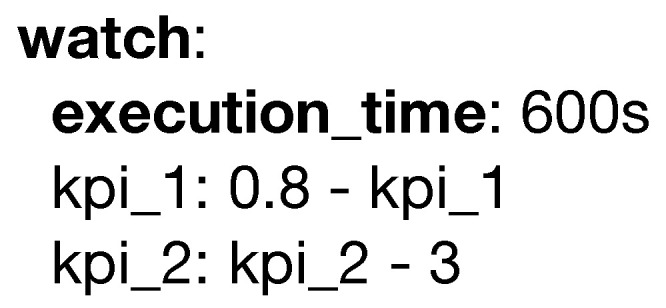
Example of the watch keyword.

A key step when moving an analytical pipeline into a production environment is guaranteeing everything performs under the specified quality constraints. In PADL, this is obtained with the **watch** keyword as seen in Figure 12. This keyword is used to define the monitorization by utilizing either language specific annotations such as *execution_time* or model specific Key PerformanceIndicators (KPI), in which the predicate represents an inequality that should be bigger than or equal to zero.

**Figure 13:**
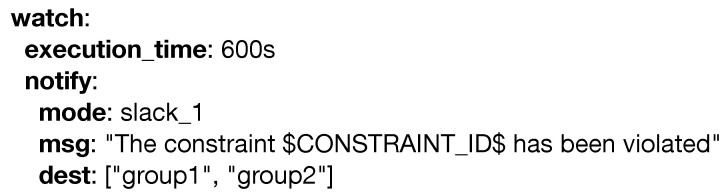
Example of the notify keyword.

If any constraint specified within the **watch** keyword is violated, an appropriate action may be specified. Currently, either the *notify* and/or the *actuate* keyword may be used. The former provides a way for sending notifications to the stakeholders when any constraint is activated. Figure 13 shows an example of its use.

**Figure 14:**
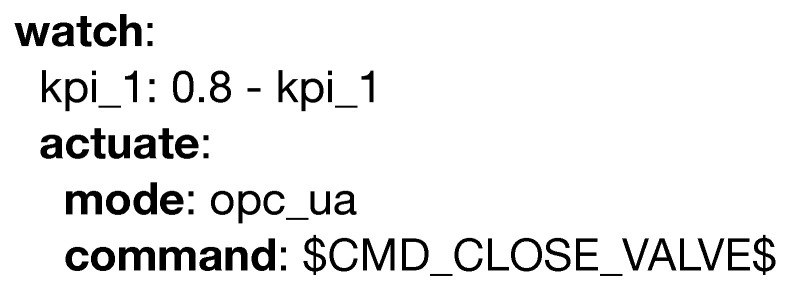
Example of the actuate keyword.

The latter, which is showcased in Figure 14, represents an automatic action taken as a reaction to a constraint violation, such as sending a command through the specified channel.

Both actions can be specified as isolated from one another or combined so, in addition to an action being taken, the stakeholders are notified through the proper channel.

## 4. PADL Implementation

As mentioned above, the deployment of artificial intelligence and machine learning models in production environments is a cumbersome process. This becomes even more obvious in smart city environments due to the variety and heterogeneity of the actors involved, whereas within a single organization, it is easier to make sure everyone is on the same page. Davenport et al. in [41] presented a survey in which they showed that even though investment keeps increasing, advances remain slow in this area. Due to this, this section aims to provide tools for alleviating this challenging process of deploying analytical pipelines in production environments.

Next, in Section 4.1, three tools developed for the use of PADL in real-life scenarios are presented. In Section 4.2, we showcase how these tools complement each other in the operationalization flow.

### 4.1. Tools

We provide three new tools that comply with the specifications described above. First, in Section 4.1.1, we offer PADLib, a library for facilitating the use of PADL in new and existing projects. In Section 4.1.2, we showcase a Command Line utility for a better integration with continuous integration and delivery pipelines, and in Section 4.1.3, we present Web Lint, which facilitates the use of PADL in the early stages of the development. The source code for these tools is publicly available on GitHub [38].

#### 4.1.1. PADLib

As part of this research, we developed a library to facilitate the use of PADL in AI and ML projects. It is a convenient way to start using the language without having to parse the definition from scratch and provides additional utilities such as the validation of the incoming file and a ready-to-use pipeline object.

The technological implementation of the specifications, described in Section 3, was developed using Python 3. The reasoning behind this decision is that Python is a highly regarded programming language in the fields of artificial intelligence and machine learning. This language has evolved from mainly being used for fast prototyping to becoming a fully functional programming language with richly featured analytic libraries (e.g., Numpy ([42]), pandas ([43]), scikit-learn ([44])) that are very appropriate for mature projects.

**Figure 15:**
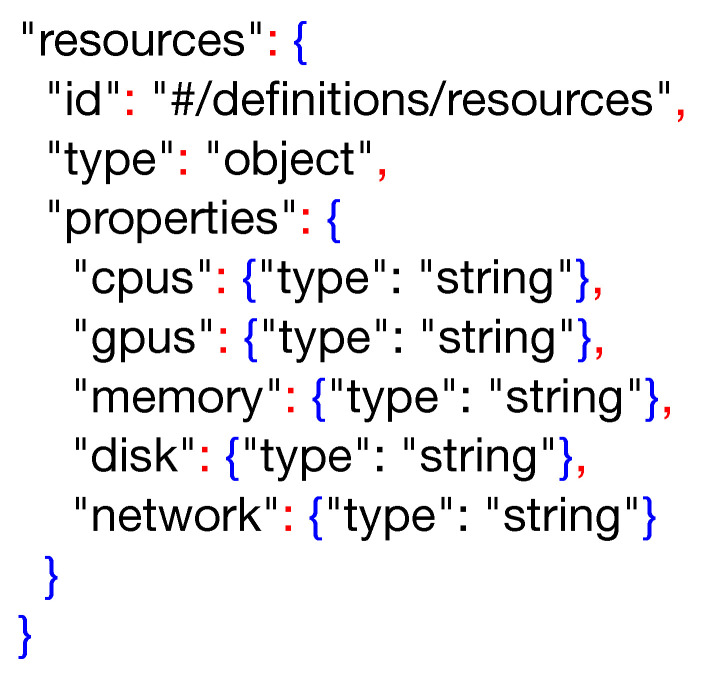
A snippet of the JSON schema definition for PADL language.

On the other hand, a PADL document is defined using the YAML format, which is very appropriate for being written and modified manually. However, to the best of the authors’ knowledge, there is no mature tool for defining the schema of a YAML document. Given that it is straightforward to transform a JSON document to YAML and vice versa, we utilize JSON Schema [45], which is a vocabulary that allows the annotation and validation of documents, as the preferred method for the description and validation of PADL documents. A snippet of this schema is shown in Figure 15.

The entire definition of PADL using JSON Schema can be found in the GitHub repository mentioned above. For the sake of brevity, only a snippet corresponding to the **resources** keyword is showcased in the listing above. This keyword has been defined as an object, which is comprised of four different entities; all of them are of the *string* type. A violation of any of these definitions would flag an error while trying to validate the document.

**Figure 16:**
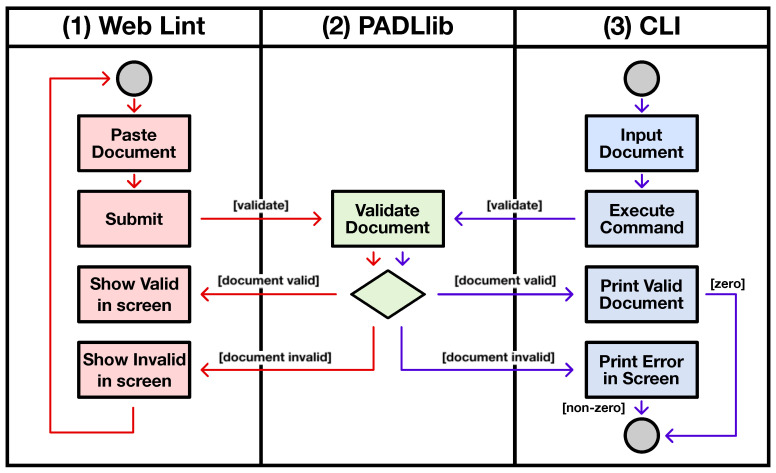
Activity diagram showcasing the interaction of the PADL library with the Web Lint and Command Line (CLI) utilities.

This schema combined with Python was used for developing the PADL API. The purpose of this API is to facilitate the use of PADL documents within artificial intelligence and machine learning projects. It provides a validation mechanism for PADL documents and parses the document into a dictionary to be easily consumed afterwards. In order to validate this development, two utilities are released within the same repository, as can be seen in Figure 16. A (1) web application (Web Lint), and a (3) Command Line utility (CLI).

Any project willing to integrate PADL into their workflow should follow a similar flow as these two tools. The invocation of the validation method becomes in (2) PADLib validating the document against the schema. Next, the library specifies whether the document is valid, indicating the cause of the error if the validation fails. If it succeeds, it will also return a dictionary with the entities of the document.

#### 4.1.2. Command Line Utility

The first tool developed using PADLib is the Command Line utility. The reasoning behind this tool is maintaining the validity of PADL documents across the development flow. This tool takes a PADL document as an input, and it validates that the file is properly formatted against the schema provided within the API. It solves two different use cases: (i) a user can utilize this tool to manually validate documents, and to this end, it shows an appropriate message if the document is correct or the corresponding error message if the document contains any error; (ii) the application returns a zero or a non-zero value whether the input document is valid or not; hence, it is an appropriate tool to validate analytical pipelines in continuous integration and deployment environments. This paves the way to a more successful deployment of analytical models in production environments.

**Figure 17:**
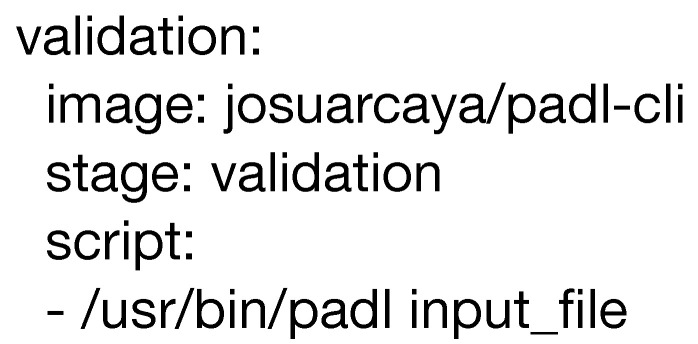
A snippet of the JSON schema defined for PADL language.

To this end, we offer a Docker image ([46]) with a stable version of the PADL Command Line utility to be used in continuous integration and deployment environments. In addition, the following snippet is a working example of the tool in conjunction with Travis-CI ([47]), which is a continuous integration and deployment server, and can be integrated into existing CI/CDpipelines. A complete example of the integration of this tool into a CI/CD pipeline can be found on GitHub, and the corresponding example can be found in Figure 17.

#### 4.1.3. Web Lint

The second tool that uses the API is a web application specifically developed for manually validating PADL documents. We identified the popularity of similar tools such as JSONlint ([48]) and YAMLlint ([49]). These tools have been widely adopted by developers in order to highlight the problems in JSON and YAML documents and lessen the complexity of using these interchangeable formats; hence, we provide an analog utility for PADL. The motivation behind such a tool is that data scientists can be certain that the pipeline they are tailoring is mature enough to be submitted to the development or production environment.

**Figure 18:**
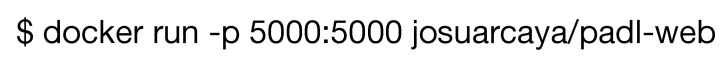
Docker command needed for running padl web.

Similarly to the Command Line tool, we provide a Docker image ([50]) that can be used locally. This image can be deployed with the command in Figure 18.

**Figure 19:**
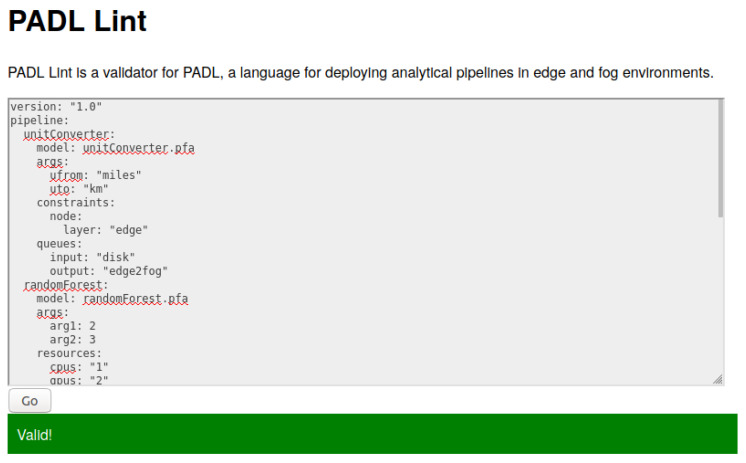
Screenshot of the Web Lint utility validating a PADL document.

Figure 19 showcases a screenshot of this tool being used for validating a document. Web Lint is designed to be straightforward to use. A wide text area preceded by a brief introduction in which the pipeline can be introduced covers the screen. Pressing the Go button will yield whether the pipeline is correct with the Valid! message highlighted in green or presents some issues that must be corrected with the Not Valid message highlighted in red.

### 4.2. Delivery Flow

The purpose of the above implementations is to provide a means for the deployment in production environments of artificial intelligence and machine learning pipelines.

**Figure 20:**
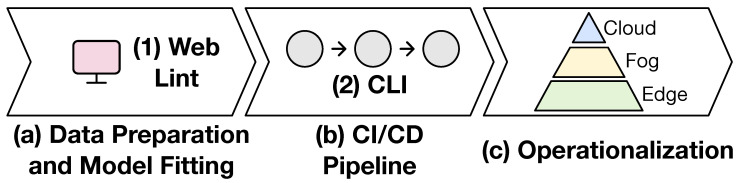
Integration of the PADLib, CLI, and Web Lint tools in the delivery flow.

Figure 20 showcases the interaction of these tools in the analytic development and deployment life cycle. In (a), the data scientist develops and tests the pipeline locally and integrates PADLibin order to utilize PADL within the pipeline. Given that the definition of this pipeline is made by hand, he/she can make use of (1) Web Lint to make sure the definition is correct, without having to interact with any programming language at all. Then, the (2) CLI becomes particularly useful in (b), the continuous integration and delivery pipeline, in order to provide a very high confidence in the production deployment. Finally, in (c), the data scientists can deploy their pipelines in the production environment with the extra confidence of having followed the described process.

## 5. PADL in Cities

In this section, we utilize two use cases from independent authors as validation for the specifications defined in Section 3 and the implementation presented in Section 4. In Section 5.2, PADL serves as the definition and deployment language for the analytical pipeline, and it complements the serious game proposed by the authors. Next, in Section 5.3, PADL complements the operationalization scenario the authors propose.

### 5.1. Use Case Selection

In this subsection, we explain the reasoning behind choosing the two use cases detailed as application examples, and we introduce the evaluation context, specifying features such as the volume of data handled or the complexity of the information flows. For the selection of these use cases, we leveraged the V’s of Big Data (i.e., Volume, Variety, Velocity).

- Flood control: In this use case, the data are produced by many sensors (high volume) measuring the water level spread across the course of the river, all of them gathering data at very low frequencies (high velocity)
- Waste management: This use case includes multiple and heterogeneous sources of information, so the variety and volume of datasets can be the main problems. Among the types of data to consider, we find: city maps, geo-referenced traffic information, garbage collection points, and other aspects such as the social or consumption information of the inhabitants, which can be used for the processes of information crossing, optimization, or calculations of indicators.

Both use cases require complex analytical processes to transform the aforementioned data into information that can assist in the decision-making, which makes them ideal for the validation of PADL.

### 5.2. Flood Control Use Case

In [51], the authors proposed the use of a serious game in the smart city of Prague, which will serve as training for the different stakeholders to react appropriately to a variety of disasters. The scenario is one in which the river Vltava, the longest river in the Czech Republic, overtops its channel’s bank and floods Prague, leading to property damage and victims. This is because the level of this river depends heavily on rainfalls taking place during its course. In order to foresee this, the level of the river is measured in different places. In addition, weather forecasting is leveraged, but due to the inherent complexity of doing so over an extended period of time, it becomes very difficult to accurately predict when and where flooding will take place.

**Figure 21:**
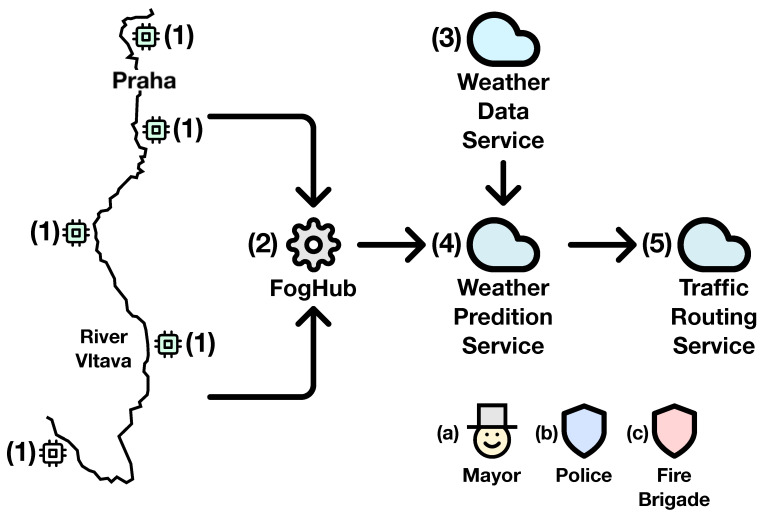
Flood control use case.

**Figure 22:**
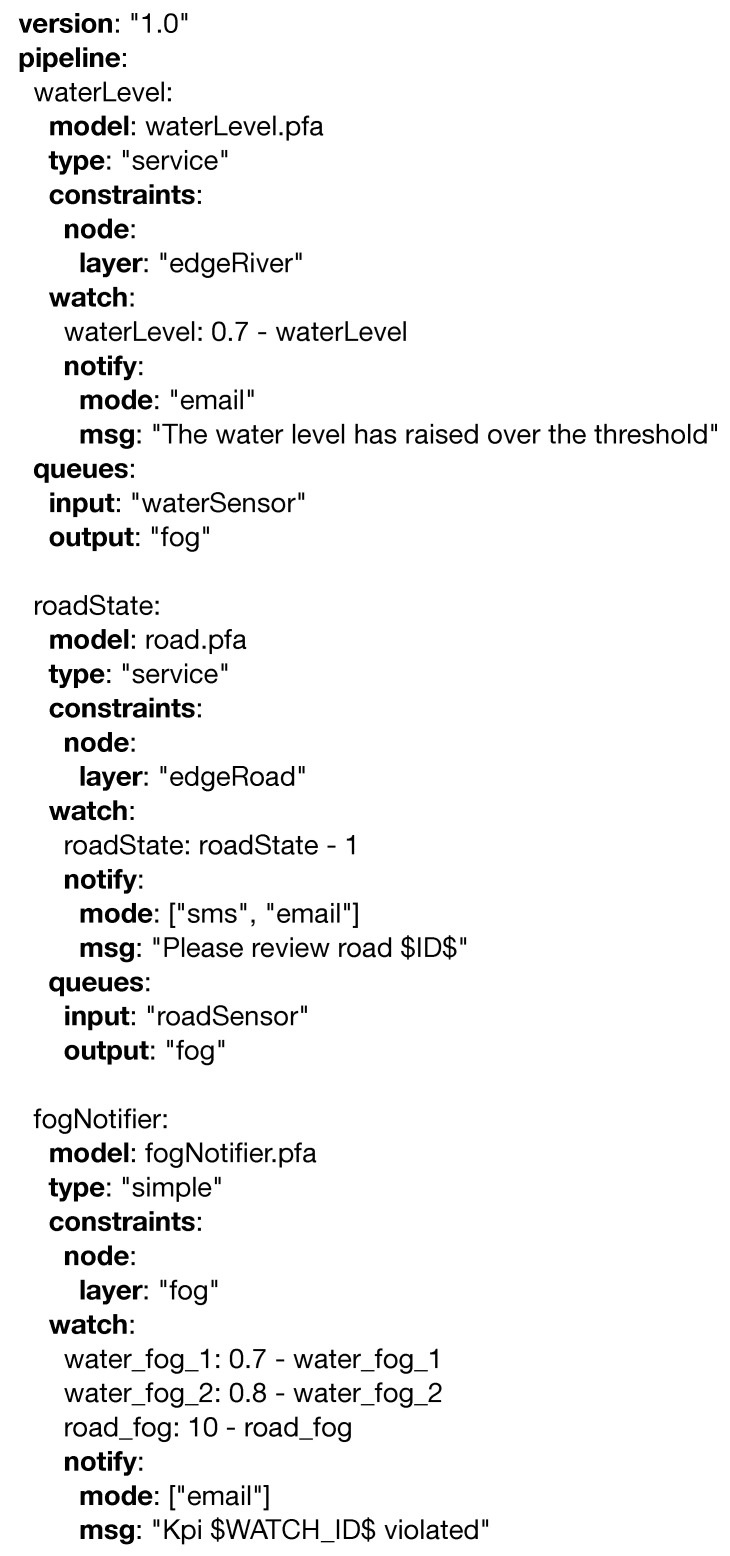
PADL document definition for the flood control usecase for the edge and fog computing layers.

This scenario is an example in which PADL excels, as it complements perfectly the serious game detailed in the paper. PADL can be used to describe the monitoring and analytical needs of the use case, and the serious game described will train the relevant stakeholders based on this information. Figure 21 showcases an overview of the relevant elements that can be managed and the relationship with the stakeholders. Firstly, the water level of the river Vltava is measured by (1) cyber physical systems installed over its course; should this level rise beyond a predefined threshold, a notification is sent to the relevant stakeholders. The actors in this scenario are defined in the serious game as follows: (i) the management team is composed of the (a) mayor and the heads of the (b) police department and (c) fire brigade; (ii) the observers, which in this scenario are (1) cyber physical systems reporting the water level in different locations, areas getting flooded, and the integrity of the removable dams; and the (iii) first responders, (b) police officers and (c) firefighters and medical staff. Secondly, if an incident (e.g., flooding) preventing a road from being used takes place, notifications are sent to the (c) fire and (b) police departments. Both of these processes operate right where data are generated, in the edge layer, and regardless of their outcome, the information is transmitted to the upper layer. The cloud layer, on the other hand, hosts three different processes. The first one, the (3) weather data service consumes data from a weather forecasting service and its duty is twofold: (i) it transforms the collected information to be better consumed by another process, and (ii) it raises alarms based on the incoming data. The second process, the (4) weather prediction serviceis fed by the (3) weather data service and the data collected in the edge layer. Within this process, a Bayesian model processes the data, and elaborated predictions and higher level notifications are produced. Such predictions can lead to the evacuation of certain areas, road closures, or event cancellations; and certain measures to prevent this such as the building of removable dams can be done in advance. Finally, the (5) traffic routing service collects information and notifications from the Weather Prediction Serviceand explores different scenarios, in which certain roads are not to be used due to the danger of flooding. This last process is utilized by rescue services (e.g., (b) fire department, (c) police department) in order to execute the assistance in the fastest and most efficient manner. Figure 22 offers the above scenario corresponding to the edge and fog layers in detail using the PADL language.

**Figure 23:**
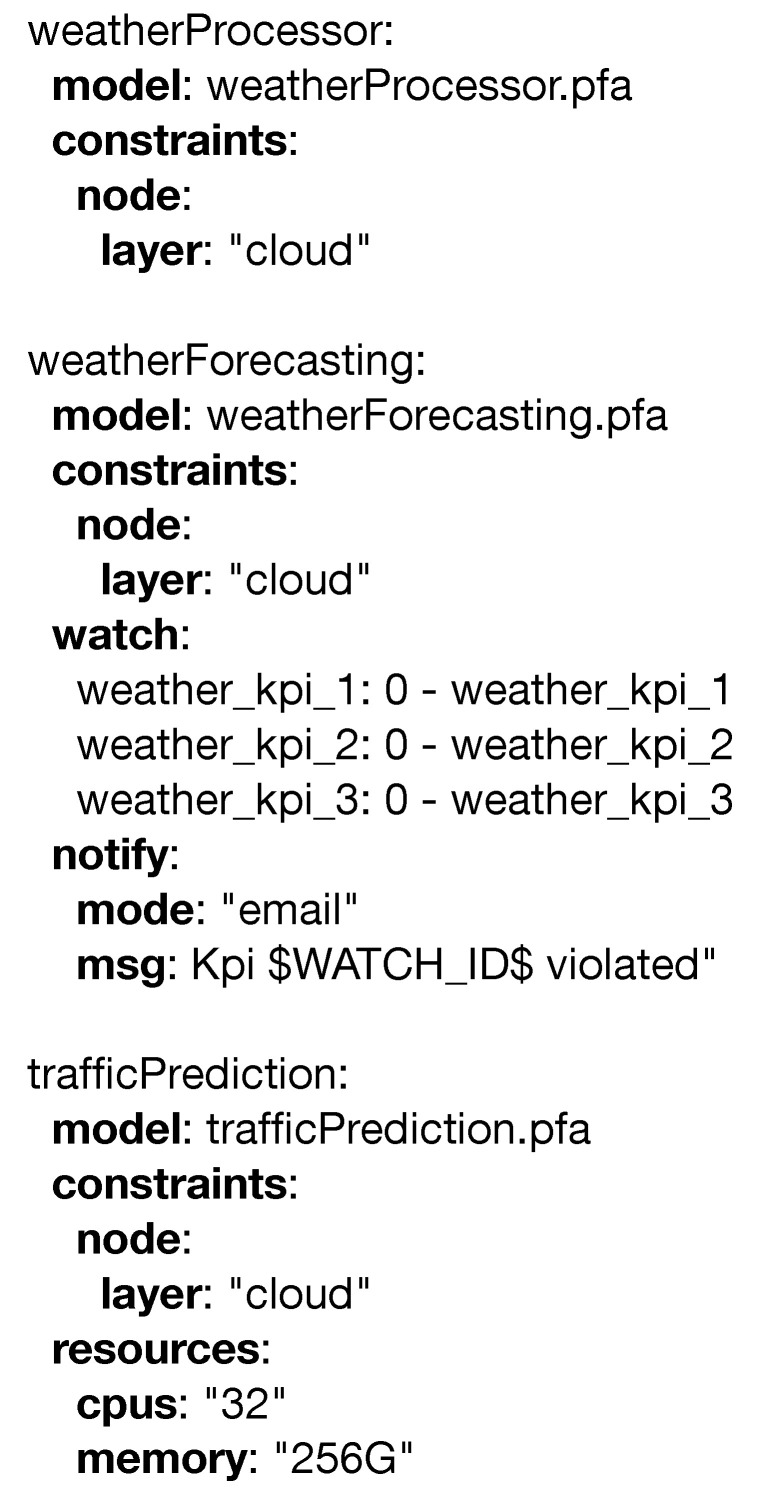
PADL document definition for the flood control usecase for the cloud computing layer.

The *waterlevel.pfa* and *road.pfa* operate in the (1) edge devices and utilize the *watch* entity to monitor and report the data coming from the sensors in case an issue is detected. *fogNotifier.pfa* operates in (2) and is able to aggregate data coming from the edge devices and evaluate more complex scenarios, such as an increase in the water flow at different points of the river. In Figure 23 the entities corresponding to the cloud layer are defined.

(3) *weatherProcessor.pfa* is in charge of collecting weather information from an external data source and sending it to the (4) *weatherForecasting.pfa* model. In this service, these data are combined with those generated in the edge and are able to make predictions, so in the case of hazardous scenarios, the relevant stakeholders can be notified (e.g., (b) police department, (c) fire brigade). Finally, *trafficPrediction.pfa* is able to produce alternative routes for the (b) police and (c) fire brigade, so in the event of a catastrophe, they can operate faster and more efficiently.

### 5.3. Waste Management Use Case

Medvedev et al. in [52] leveraged Internet of Things components such as (i) RFIDs, (ii) sensors, (iii) cameras, and (iv) actuators in a smart city environment and proposed an advanced Decision Support System (DSS) for efficient waste collection. This system incorporates data generated by the smart devices and truck drivers in real time and feeds them into a dynamic route optimization model. This model minimizes the inefficiencies of waste collection within the smart city, by providing alternative routes for the drivers, taking into account traffic jams, inaccessible waste bins, and problematic areas. The final goal of the waste collection system is to improve the quality of service for the citizens of the smart city.

**Figure 24:**
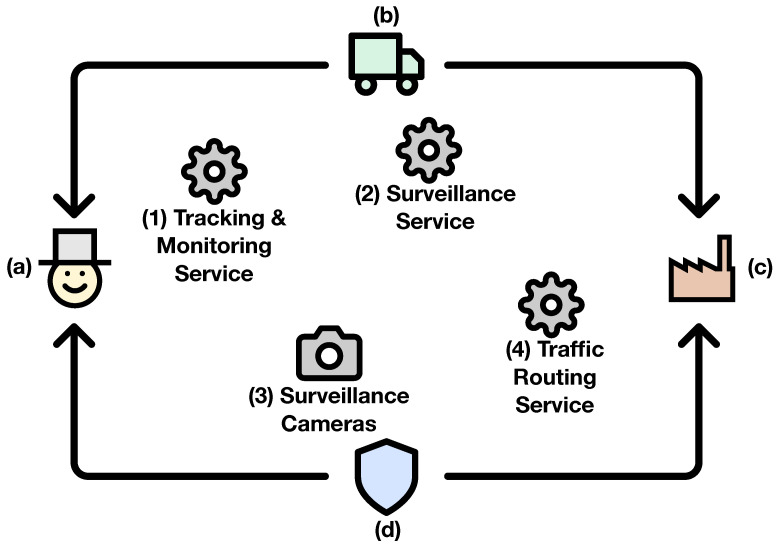
Waste management use case.

**Figure 25:**
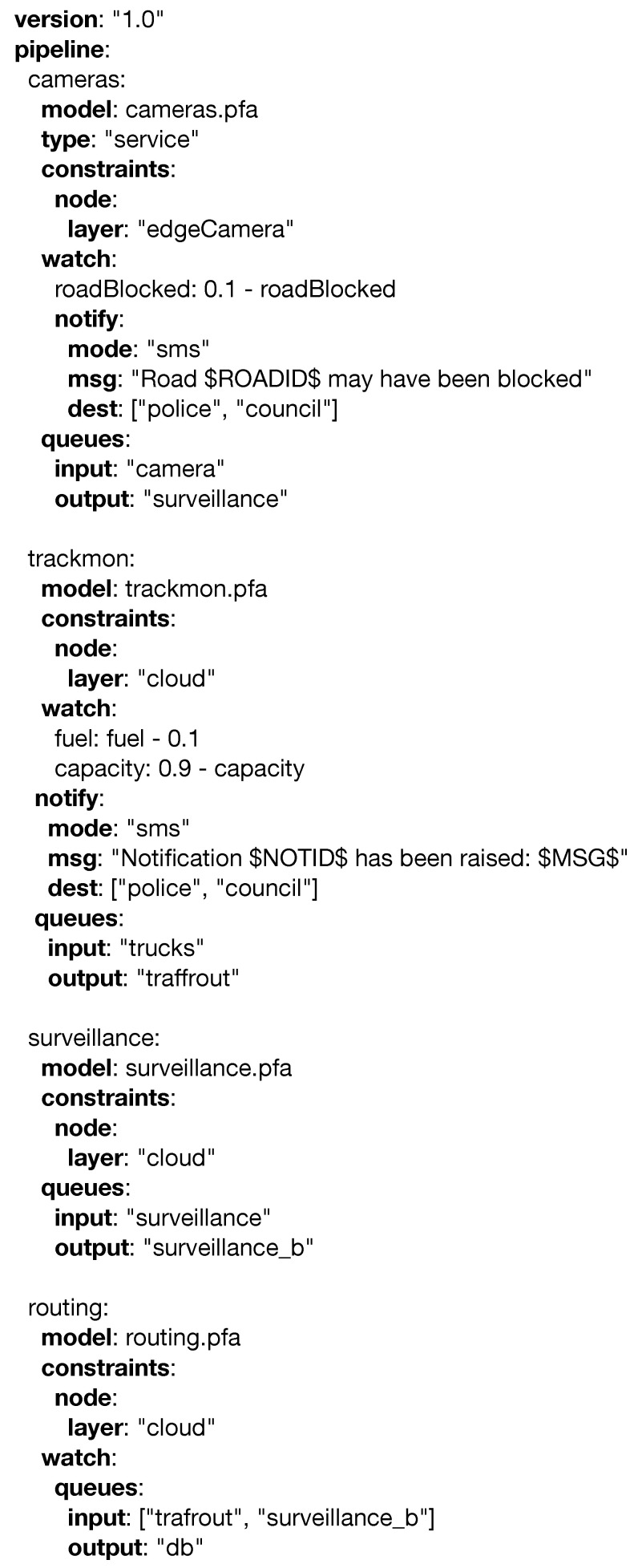
PADL document for the waste management use case.

The stakeholders in this scenario greatly benefit from the monitoring and notification capabilities of PADL: the (a) city and district administrations are interested in controlling the process of waste collection, both from a quality of service point of view (e.g., all collected cleanly and in time) and from a legal point of view (e.g., collect evidence for solving disputes); (b) waste truck organizations and drivers want to monitor and track the fleet and find alternative routes based on data gathered from IoT devices; (c) recycling factories want to send notifications based on their current needs and limitations; and the (d) police department needs to be notified if improper parking is preventing the waste bins from being emptied. These entities alongside the services interacting in this use case are represented in Figure 24. Some of these entities feed information into the system. First, Entity (b), waste trucks, use IoT devices to report their location, capacity, and fuel in real time. In addition, the drivers are able to report problems (e.g., improper parking) while collecting waste bins. This information includes video and audio information that the drivers are able to publish by using mobile devices. Entity (c), recycling factories, are able to publish their capacities or needs based on their storing or recycling desires. Finally, the ecosystem is able to process information coming from surveillance cameras and traffic and weather services. Three services comprise the analytical engine of the ecosystem. Firstly, the (1) Tracking and Monitoring Service is able to provide metrics and notifications to the *(b) Waste Organizations* so they are provided with advanced insights about their business to make better decisions. Secondly, the *(2) Surveillance Service* uses the data generated by surveillance cameras and notifies (a) the *City Administration*, (b) truck drivers, and (d) police department in case a problem (e.g., traffic jam, blocked road) is detected. Finally, the *(3) Traffic Routing Service* is able to provide dynamic routes to the (b) truck driversbased on all the information being fed into the system, hence minimizing the time needed to perform their duties. There are two entities (the *(a) City and District Administrations* and the *(d) Police Department*) that do not feed the ecosystem with data, but only interact with it by receiving notifications. The former wants to be up to date on the whereabouts of the waste recollection process in the city, whereas the latter will react to the notifications being sent by the *(2) Surveillance Service* and the *(b) Truck Drivers* in order to solve the issues being raised. Figure 25 defines the above scenario using the PADL language.

The *cameras.pfa* model operates in the edge devices and is able to notify about blocked roads to the (d) Police Department. In addition, it serves as an input for the (2) *Surveillance Service*, which performs a wider aggregation of the data generated by the cameras and is able to provide high level notifications such as traffic jams. The (1) *trackmon.pfa* model operates in the cloud and is able to get the information produced by track drivers. Finally, the (3) *routing.pfa* provides alternative routes based on all the data and insights generated by the ecosystem.

### 5.4. Use Case Discussion

This section presents the main results of the process to validate the proposed language by means of both use cases: flood control and waste management. In order to trust in the feasibility of the PADL language to represent analytic pipelines and to operate them in production environments, it is relevant to compare the definition file provided in each use case against the criteria in Table 2. In addition, a seventh criterion is analyzed regarding the complexity of the use case measured through its characterization through the three V’s of Big Data: volume, variety, and velocity. This analysis can be seen in the following table, where the use cases are presented as the rows and the evaluation criteria form the columns.

Based on our observations of these use cases’ PADL implementations, we were are encouraged by the results, showing that:

1. Criterion (1): We provide domain specific annotations for the appropriate deployment of each step in the pipeline, such as the measurement of the water level in the river in the first use case and being able to detect blocked roads with the cameras in the second one.
2. Criterion (2): Monitorization, notification, and actuation are handled separately for each model. For example, in the second use case, the language enables the chance of monitoring and reporting a low fuel level for the waste truck.
3. Criterion (3): We utilize technologies and smart devices already present in the use cases, like the ones used for reporting the river level in the water flood use case.
4. Criterion (4): All the definitions of the use case analytic pipelines are covered in the PADL snippets in Section 5.2 and Section 5.3.
5. Criterion (5): We do not require additional technologies other than the ones already in use by the city. For example, in the case of waste management, cameras are the smart devices that are mainly used.
6. Criterion (6): The different steps of the pipeline are deployed across the existing infrastructure and can be operationalized independently. For example, in the waste management use case, analytic processing is distributed over the heterogeneous infrastructure: waste trucks, cameras, cloud servers.
7. Criterion (7): The main dimensions of Big Data, volume, velocity, and variety, are highly represented in the use case. In the flood control use case, the most stringent restriction is the speed of data collection and analysis for the flooding alerts in real time. In the waste management use case, on the other hand, the volume and heterogeneity of the data sources are the greatest challenges, due to the need to cross-reference information to draw valuable conclusions that help optimize waste management.

In conclusion, PADL is an appropriate solution for the smart city domain, advancing over existing tools for similar purposes as per the evaluation criteria in Table 1. The previous analysis shows that PADL excels in defining complex analytical pipeline flows, with multiple considerations (heterogeneous data sources, multiple devices to consider, or different types of processing) and various phases (e.g., monitorization, notification, actuation).

## 6. Conclusions and Future Work

In this research, we propose a domain specific language for the definition of distributed analytical pipelines, which alleviates the burden of deploying data science projects in production environments. On one hand, it abstracts data scientists from the underlying technologies, networking challenges, and deployment specific constraints, hence letting them focus on the functionalities. On the other hand, the team in charge of the operationalization obtains a detailed description of the process for deploying the given pipeline in the production environment. Overall, PADL aims at raising the success rate of data science projects, by mitigating the challenges of deploying such projects in real-world scenarios. The use of modern mechanisms to provide these pipelines with monitorization, notification, and actuation capabilities promote the utilization of this language in the emerging IoT paradigm. In order to do so, we provide tools that cover the operationalization of ML (Machine Learning) and AI (Artificial Intelligence) projects over its different stages and lighten the burden of deploying these projects in edge, fog, and cloud environments successfully. As part of this effort, we analyze two use cases from independent research papers in a smart city context and elucidate how PADL can be used to implement the analytical needs that complement them in order to provide an integrated solution. Both use cases demonstrate the benefits that PADL offers in the smart city domain, and unlike the rest of the alternatives under study, it has been shown to comply with all the criteria defined in Table 1. The source code with the language specification and the developed tools are provided by Díaz-de-Arcaya et al. in [38].

As for the future work, we consider two research directions: firstly, to promote PADL as the standard for the definition of analytical pipelines leading to the development or evolution of other tools or utilities; secondly, to increase the expressiveness of PADL with new functionalities and characteristics spanning a broader range of the analytical life cycle. In the first line of study, our idea is to utilize PADL alongside the definition of a given infrastructure to implement the orchestrator appearing in Figure 1; in this way obtaining the best possible deployment for a given analytical pipeline. Furthermore, the emergence of public cloud providers in conjunction with infrastructure-as-code technologies gives us the possibility of generating the necessary infrastructure on demand.

## Figures and Tables

**Table 1:** Technologies for deploying analytical pipelines in production environments.

	Deployment Awareness	Analytics Oriented	Technology Agnostic	Text Based	Small Technological Footprint	Multilayer Awareness
PADL	f	f	f	f	f	f
PFA	n	f	n	f	n	n
PMML	n	f	n	f	n	n
MLflow	s	f	s	n	f	n
ML.NET	s	f	n	n	n	n
Kubernetes	f	n	n	f	n	n
Docker Swarm	f	n	s	f	s	n
Stratum	s	f	s	n	s	n
DEEP-Hybrid-DataCloud	s	f	s	n	s	n

f, full support; n, not supported; s, to some extent.

**Table 2:** PADL functionalities following the criteria of Table 1 validated against the use cases.

	Deployment Awareness	Analytics Oriented	Technology Agnostic	Text Based	Small Technological Footprint	Multilayer Awareness	Big Data Dimensions
Flood control	y	y	y	y	y	y	Volume and Velocity
Waste Management	y	y	-	y	y	y	Volume and Variety

y, validated; n, not validated.
